# Crystal structure, optical property and Hirshfeld surface analysis of bis­[1-(prop-2-en-1-yl)-1*H*-imidazol-3-ium] hexa­chlorido­stannate(IV)

**DOI:** 10.1107/S2056989020012177

**Published:** 2020-09-08

**Authors:** Hela Ferjani

**Affiliations:** a Chemistry Department, College of Science, IMSIU (Imam Mohammad Ibn Saud Islamic University), Riyadh 11623, Kingdom of Saudi Arabia

**Keywords:** chlorido­stannate(IV), 1-(prop-2-en-1-yl)-1*H*-imidazole, Hirshfeld surface, fingerprint plots, optical absorption., crystal structure

## Abstract

A new 0D organic–inorganic hybrid material bis­[1-(prop-2-en-1-yl)-1*H*-imidazol-3-ium] hexa­chlorido­stannate(IV), (C_6_H_9_N_2_)_2_[SnCl_6_], has been prepared and characterized by single-crystal X-ray diffraction, Hirshfeld surface analysis and UV–visible spectroscopy. The structure consists of isolated [SnCl_6_]^2−^ octa­hedral anions separated by layers of organic 1-(prop-2-en-1-yl)-1*H*-imidazol-3-ium cations. The 1-(prop-2-en-1-yl) fragment in the organic cation exhibits disorder over two sets of atomic sites having occupancies of 0.512 (9) and 0.488 (9). The crystal packing of the title compound is established by inter­molecular N/C–H⋯Cl hydrogen bond and π– π stacking inter­actions. Hirshfeld surface analysis employing three-dimensional mol­ecular surface contours and two-dimensional fingerprint plots has been used to analyse the inter­molecular inter­actions present in the structure. The optical properties of the crystal were studied using UV–visible absorption spectroscopy, showing one intense band at 208 nm, which is attributed to π–π* transitions in the cations.

## Abstract 2

The title structure consists of isolated [SnCl_6_]^2−^ octa­hedral anions separated by layers of organic 1-(prop-2-en-1-yl)-1*H*-imidazol-3-ium (C_6_H_9_N_2_)^+^ cations. The crystal packing features inter­molecular N—H⋯Cl and C—H⋯Cl hydrogen bonds and π–π stacking inter­actions.

## Chemical context   

Tin(IV) halide organic–inorganic hybrid compounds are significant materials because of their inter­esting structural topologies and their wide range of optical applications such as luminescence, non-linear activity and semiconductivity (Hajji *et al.*, 2016 ▸, 2019 ▸; BelhajSalah *et al.*, 2018 ▸). As part of a continuing search of new organic–inorganic hybrid compounds such as Bi_2_Cl_10_^4−^ (Ferjani & Boughzala, 2018 ▸; Ferjani *et al.*, 2020 ▸) and Hg_2_Cl_6_ (Garci *et al.*, 2019 ▸), the synthesis and characterization of a new hybrid material, bis­[1-(prop-2-en-1-yl)-1*H*-imidazol-3-ium] hexa­chlorido­stan­nate(IV), (C_6_H_9_N_2_)_2_[SnCl_6_] is reported.
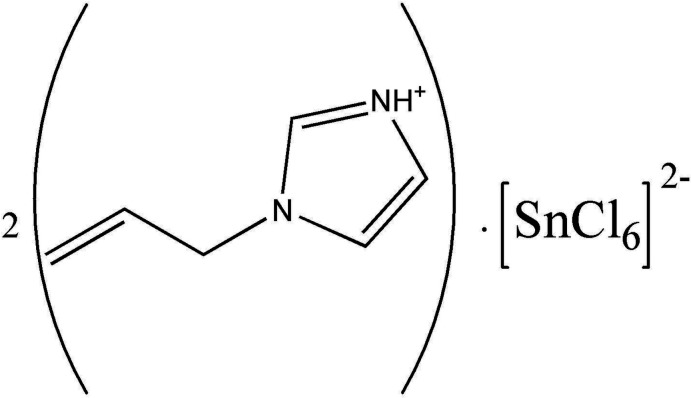


Imidazole was chosen as the organic cation because the resulting complexes show inter­esting structural, chemical and physical properties significant for photoluminescence, magnetism, ferroelectricity, and conductivity (Tritt-Goc *et al.*, 2019 ▸; Babar *et al.*, 2019 ▸; Ishak *et al.*, 2019 ▸). The Hirshfeld surface analysis was performed to completely characterize the inter­molecular inter­actions and explain the crystalline architecture. Moreover, the UV–visible spectrum was also investigated.

## Structural commentary   

**Figure 1:**
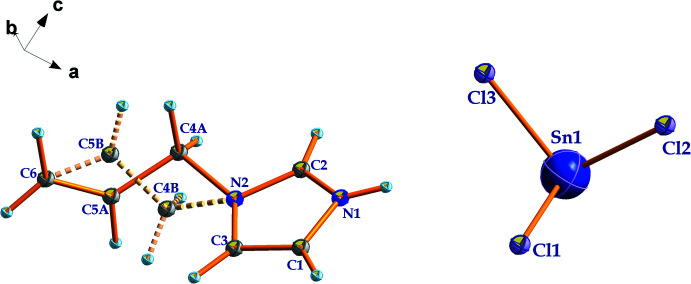
The asymmetric unit of (C_6_H_9_N_2_)_2_[SnCl_6_], showing the atom-labelling scheme and the disorder of the allyl group with occupancies of 0.512 (9) (solid bonds) and 0.488 (9) (broken bonds).

**Figure 2:**
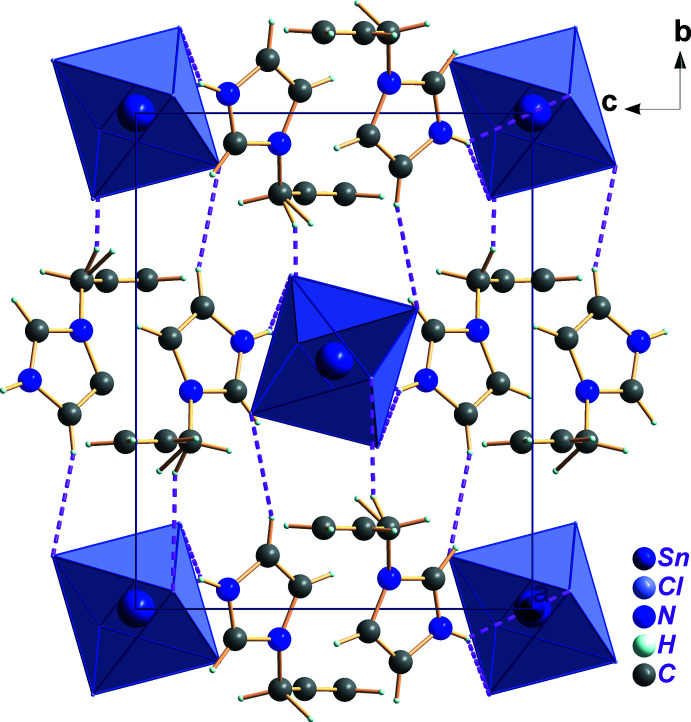
Crystal packing of (C_6_H_9_N_2_)_2_[SnCl_6_] viewed along the *a* axis, showing the N—H⋯Cl and C—H⋯Cl hydrogen bonds (dashed lines).

The asymmetric unit of (C_6_H_9_N_2_)_2_[SnCl_6_] contains one (C_6_H_9_N_2_)^+^ cation and one half of an [SnCl_6_]^2−^ anion (Fig. 1 ▸). The Sn^IV^ atom is located on a special position of site symmetry 2_1_/*n* (crystallographic center of inversion) and is coordinated by six chlorine atoms in an octa­hedral geometry. The hexa­chloro­stannate(IV) octa­hedron is nearly perfect with Sn—Cl bond lengths ranging from 2.4136 (6) to 2.4363 (6) Å and Cl—Sn—Cl bond angles between 88.44 (3) and 180°. These bond lengths and angles are in good agreement with those observed in similar compounds based on hexa­chloro­stannate(IV) (van Megen *et al.*, 2013 ▸; Zhou *et al.*, 2012 ▸; Rademeyer *et al.*, 2007 ▸). The organic (C_6_H_9_N_2_)^+^ cations are related to each other by 2_1_/*n* symmetry elements. The overall negative charges in the structure are counter-balanced by the protonated 1-(prop-2-en-1-yl)-1*H*-imidazol-3-ium cations (Fig. 1 ▸). As usual, this aromatic amine is protonated at the N1 atom. The C=C and ring C—N bond lengths vary from 1.253 (8) to 1.307 (5), and 1.265 (6) to 1.349 (5) Å, respectively, which agree well with those in homologous materials involving 1-(prop-2-en-1-yl)-1*H*-imidazole (Ferjani, 2020 ▸; Parshina *et al.*, 2019 ▸). The crystal structure can be described as an organization of organic–inorganic layers, which propagate along the *a* axis at *y* = 0 and *y* = 1/2 (Fig. 2 ▸). These layers are formed by [SnCl_6_]^2−^ octa­hedra and (C_6_H_9_N_2_)^+^ organic cations.

## Supra­molecular features   

**Figure 3:**
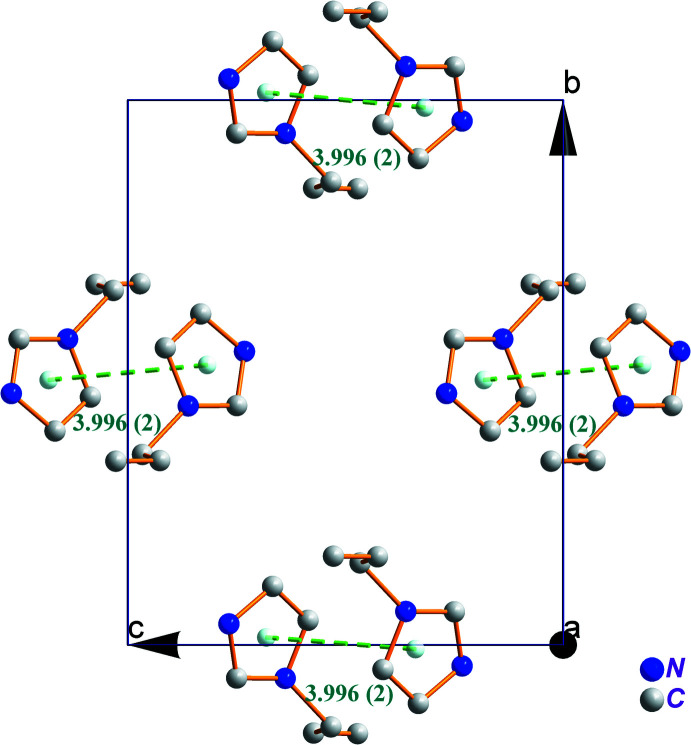
The π–π inter­actions between organic cations in (C_6_H_9_N_2_)_2_[SnCl_6_]. H atoms are omitted for clarity.

The cohesion and stabilization of the title structure is ensured by N—H⋯Cl and C—H⋯Cl hydrogen bonds between the NH^+^ unit of 1*H*-imidazol-3-ium as the donor group and the chlorine atoms of the [SnCl_6_]^2−^ octa­hedron as acceptor with H⋯Cl lengths ranging between 2.67 and 2.98 Å (Fig. 2 ▸ and Table 1 ▸). Additional stabilization is provided by weak π–π stacking inter­actions between 1*H*-imidazol-3-ium rings with a centroid-to-centroid distance of 3.996 (2) Å (Fig. 3 ▸).

## Hirshfeld surface analysis   

**Figure 4:**
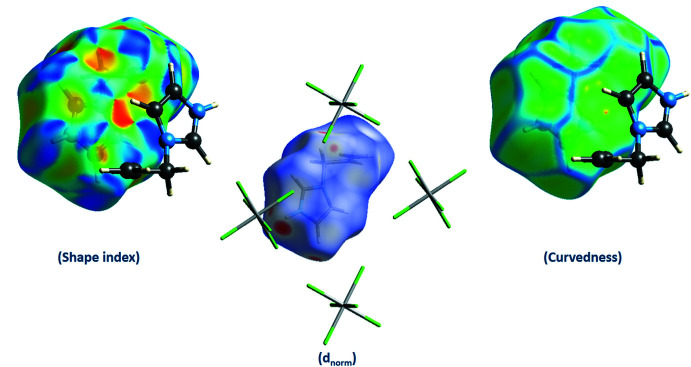
View of the Hirshfeld surfaces for (C_6_H_9_N_2_)_2_[SnCl_6_] mapped over shape-index, *d*_norm_ and curvedness.

The Hirshfeld surface analysis (Spackman & Jayatilaka, 2009 ▸) was performed and the associated 2D fingerprint plots (McKinnon *et al.*, 2007 ▸) generated using *Crystal Explorer 17* (Turner *et al.*, 2017 ▸). The Hirshfeld surface was calculated using a standard (high) surface resolution with the three-dimensional (3D) *d*_norm_ surface plotted over a fixed colour scale mapped over the range −0.208 (red) to 1.180 (blue) a.u. The *d*_norm_ mapping indicates that strong hydrogen-bonding inter­actions, such as N—H⋯Cl hydrogen bonding between chlorine atoms and imidazolium groups and C—H⋯Cl hydrogen bonding between chlorine atoms and the hydrogen atoms of the 1-(prop-2-en-1-yl) groups, appear to be the primary inter­actions in the structure, seen as a bright-red area in the Hirshfeld surface (Fig. 4 ▸).

A shape-index map of the title compound was calculated in the range −0.995 to 0.996 a.u. (Fig. 4 ▸). The convex blue regions on the shape-index symbolize hydrogen-donor groups and the concave red regions symbolize hydrogen-acceptor groups. π–π inter­actions are generally indicated by adjacent red and blue triangles on the shape-index map of the Hirshfeld surface.

A curvedness map of the title compound was generated in the range −3.411 to 0.368 a.u. (Fig. 4 ▸). The large flat region of green around the rings delineated by a blue outline on the Hirshfeld surface plotted over curvedness refer to the π–π stacking inter­actions.

**Figure 5:**
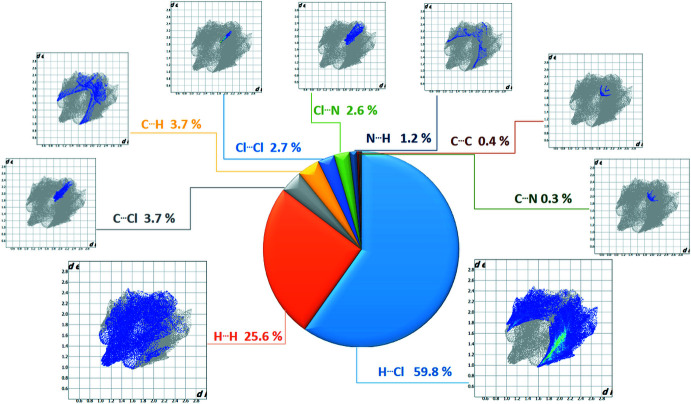
Two-dimensional fingerprint plots for the title compound showing the contributions of different types of inter­actions.

The overall 2D fingerprint plot for all contacts are shown in Fig. 5 ▸, together with their relative contributions to the Hirshfeld surface. The 2D fingerprint plots show that the dominant inter­molecular H⋯Cl (N/C-H⋯Cl) and H⋯H inter­actions contribute 59.8% and 25.6%, respectively, to the overall crystal packing. The fingerprint plot of H⋯Cl contacts, which represent the largest contribution to the Hirshfeld surfaces (59.8%), shows two large spikes highly concentrated at the edges, having almost the same *d*_e_ + *d*_i_ = 2.7 Å (Fig. 5 ▸). The H⋯H inter­actions appear as the next largest region of the fingerprint plot (25.6%), and have a distinct pattern with a minimum value of *d*_e_ = *d*_i_ = 1 Å (Fig. 5 ▸). Apart from these above, C⋯Cl, C⋯H, Cl⋯Cl, Cl⋯N, N⋯H, C⋯C, and C⋯N inter­actions were observed, which are summarized in Fig. 5 ▸.

## Database survey   

In the Cambridge Structural Database (Version 5.40, November 2019; Groom *et al.*, 2016 ▸), eight structures of transition-metal coordination compounds with the 1-allyl­imidazole ligand are reported. The environment for the central ion in the [*ML*_6_]^2+^ ion is provided by the nitro­gen atoms of six imidazole rings (Kurdziel & Glowiak, 2000 ▸; Kurdziel & Glowiak, 1998 ▸) or by other ligands with the imidazole rings (Glowiak & Kurdziel, 2000 ▸; Curtis *et al.* 2008 ▸; Kurdziel & Glowiak, 1998 ▸; Li & Liu, 2010 ▸). However, there is no structure reported of a post-transition-metal complex with 1-allyl­imidazole as ligand. One bis­muth complex with 1-allyl­imidazole (C_6_H_9_N_2_)_4_[Bi_4_I_16_]·2H_2_O has been recently determined by Ferjani (2020 ▸), but is not yet available in the CSD. This and the title structure have the same monoclinic crystallographic *P*2_1_/*n* symmetry. However, one has two cations in the unit cell and the other has only one. The half anionic cluster in the asymmetric unit sits on a crystallographic inversion center.

## UV–visible spectroscopy   

**Figure 6:**
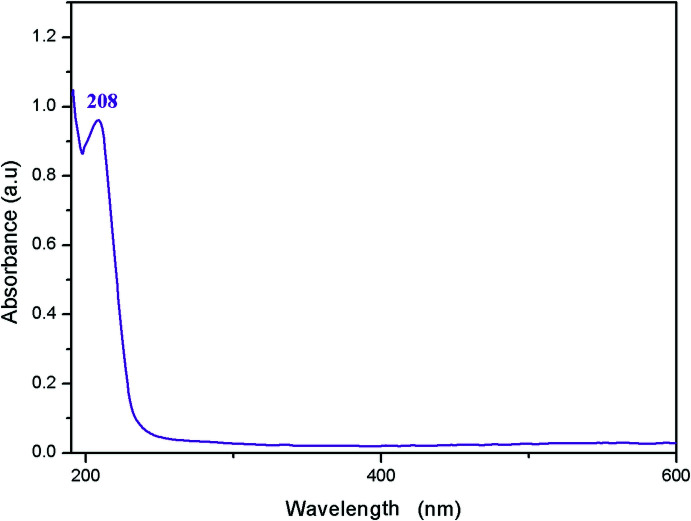
UV–visible spectrum of the title compound.

Optical absorption (OA) of the title compound was measured at ambient temperature in water. The experimental UV–visible absorption spectrum of the title compound is shown in Fig. 6 ▸. It shows one intense absorption band at 208 nm. According to a similar compound studied previously (Maalaoui *et al.*, 2012 ▸; Lassoued *et al.*, 2017 ▸; Hermi *et al.*, 2020 ▸; Mathlouthi *et al.*, 2017 ▸), we assign this band to π–π* transitions within the (C_6_H_9_N_2_)^+^ organic cations.

## Synthesis and crystallization   

The title compound was prepared by dissolving 0.34 g (1 mmol) of 1-allyl­imidazole [1-(prop-2-en-1-yl)-1*H*-imidazole] and 0.3 g (2 mmol) of tin(II) chloride in 10 ml of concentrated (37%) hydro­chloric acid. The mixture was stirred with heating and then kept at room temperature. Three days later, colourless single crystals suitable for structural determination were obtained.

## Refinement   

Crystal data, data collection and structure refinement details are summarized in Table 2 ▸. The disordered 1-(prop-2-en-1-yl) fragment in the organic cation was refined by splitting atoms C4 and C5 over two positions (C4*A*, C4*B*) and (C5*A*, C5*B*) with occupancy factors of 0.512 (9) and 0.488 (9). Geometrical restraints (SADI) on bond lengths were applied. H atoms were located in difference-Fourier maps but introduced in calculated positions and treated as riding on their parent atoms, with C—H = 0.93 and 0.97 Å, N—H = 0.86 Å with *U*_iso_(H) = 1.2*U*_eq_(C, N).

## Supplementary Material

Crystal structure: contains datablock(s) I. DOI: 10.1107/S2056989020012177/vm2239sup1.cif

Structure factors: contains datablock(s) I. DOI: 10.1107/S2056989020012177/vm2239Isup3.hkl

CCDC reference: 2021940

Additional supporting information:  crystallographic information; 3D view; checkCIF report

## Figures and Tables

**Table 1:** Hydrogen-bond geometry (Å, °)

*D*—H⋯*A*	*D*—H	H⋯*A*	*D*⋯*A*	*D*—H⋯*A*
N1—H1*A*⋯Cl2	0.86	2.67	3.399 (4)	144
N1—H1*A*⋯Cl1	0.86	2.81	3.485 (4)	136
C2—H2⋯Cl3^i^	0.93	2.79	3.672 (4)	158
C3—H3⋯Cl3^ii^	0.93	2.90	3.790 (4)	160
C4*A*—H4*A*1⋯Cl2^iii^	0.97	2.89	3.716 (9)	144
C4*A*—H4*A*1⋯Cl1^iv^	0.97	2.94	3.743 (10)	141
C4*B*—H4*B*1⋯Cl1^v^	0.97	2.98	3.702 (10)	133
C4*B*—H4*B*2⋯Cl1^iv^	0.97	2.80	3.590 (7)	139
C5*B*—H5*B*⋯Cl2^vi^	0.93	2.97	3.766 (11)	145

Symmetry codes: (i) 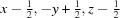; (ii) 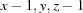; (iii) 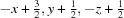; (iv) 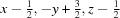; (v) 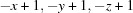; (vi) 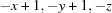.

**Table 2:** Experimental details

Crystal data	
Chemical formula	(C_6_H_8_N_2_)_2_[SnCl_6_]
*M*_r_	547.68
Crystal system, space group	Monoclinic, *P*2_1_/*n*
Temperature (K)	298
*a*, *b*, *c* (Å)	9.3953 (5), 11.7817 (6), 9.8243 (5)
β (°)	106.547 (4)
*V* (Å^3^)	1042.44 (10)
*Z*	2
Radiation type	Mo *K*α
μ (mm^−1^)	2.00
Crystal size (mm)	0.71 × 0.66 × 0.42

Data collection	
Diffractometer	Stoe IPDS2
Absorption correction	Integration (*X-RED32*; Stoe & Cie, 2002 ▸)
*T*_min_, *T*_max_	0.311, 0.358
No. of measured, independent and observed [*I* > 2σ(*I*)] reflections	12972, 4294, 3326
*R*_int_	0.035
(sin θ/λ)_max_ (Å^−1^)	0.791

Refinement	
*R*[*F* ^2^ > 2σ(*F* ^2^)], *wR*(*F* ^2^), *S*	0.039, 0.086, 1.14
No. of reflections	4294
No. of parameters	126
No. of restraints	5
H-atom treatment	H-atom parameters constrained
Δρ_max_, Δρ_min_ (e Å^−3^)	0.65, −0.93

Computer programs: *X-AREA* and *X-RED32* (Stoe & Cie, 2002 ▸), *SHELXT* (Sheldrick, 2015*a* ▸), *SHELXL2014/7* (Sheldrick, 2015*b* ▸), *DIAMOND* (Brandenburg, 2006 ▸) and *publCIF* (Westrip, 2010 ▸).

## supplementary crystallographic information

### Crystal data

**Table:**

(C_6_H_8_N_2_)_2_[SnCl_6_]	*F*(000) = 536
*M**_r_* = 547.68	*D*_x_ = 1.745 Mg m^−^^3^
Monoclinic, *P*2_1_/*n*	Mo *K*α radiation, λ = 0.71073 Å
*a* = 9.3953 (5) Å	Cell parameters from 19596 reflections
*b* = 11.7817 (6) Å	θ = 2.3–34.4°
*c* = 9.8243 (5) Å	µ = 2.00 mm^−^^1^
β = 106.547 (4)°	*T* = 298 K
*V* = 1042.44 (10) Å^3^	Prism, colorless
*Z* = 2	0.71 × 0.66 × 0.42 mm

### Data collection

**Table:**

Stoe IPDS2 diffractometer	4294 independent reflections
Radiation source: sealed X-ray tube, 12 x 0.4 mm long-fine focus	3326 reflections with *I* > 2σ(*I*)
Detector resolution: 6.67 pixels mm^-1^	*R*_int_ = 0.035
rotation method scans	θ_max_ = 34.2°, θ_min_ = 2.7°
Absorption correction: integration (X-RED32; Stoe & Cie, 2002)	*h* = −11→14
*T*_min_ = 0.311, *T*_max_ = 0.358	*k* = −17→18
12972 measured reflections	*l* = −15→15

### Refinement

**Table:**

Refinement on *F*^2^	Hydrogen site location: inferred from neighbouring sites
Least-squares matrix: full	H-atom parameters constrained
*R*[*F*^2^ > 2σ(*F*^2^)] = 0.039	*w* = 1/[σ^2^(*F*_o_^2^) + (0.0288*P*)^2^ + 0.5447*P*] where *P* = (*F*_o_^2^ + 2*F*_c_^2^)/3
*wR*(*F*^2^) = 0.086	(Δ/σ)_max_ < 0.001
*S* = 1.14	Δρ_max_ = 0.65 e Å^−^^3^
4294 reflections	Δρ_min_ = −0.93 e Å^−^^3^
126 parameters	Extinction correction: SHELXL-2014/7 (Sheldrick 2014, Fc^*^=kFc[1+0.001xFc^2^λ^3^/sin(2θ)]^-1/4^
5 restraints	Extinction coefficient: 0.0276 (10)

### Special details

**Table:**

Geometry. All esds (except the esd in the dihedral angle between two l.s. planes) are estimated using the full covariance matrix. The cell esds are taken into account individually in the estimation of esds in distances, angles and torsion angles; correlations between esds in cell parameters are only used when they are defined by crystal symmetry. An approximate (isotropic) treatment of cell esds is used for estimating esds involving l.s. planes.

### Fractional atomic coordinates and isotropic or equivalent isotropic displacement parameters (Å^2^)

**Table:**

	*x*	*y*	*z*	*U*_iso_*/*U*_eq_	Occ. (<1)
Sn1	1.0000	0.5000	0.5000	0.03804 (7)	
Cl2	0.87600 (8)	0.32725 (6)	0.39099 (8)	0.05747 (17)	
Cl3	1.12514 (8)	0.39362 (6)	0.70986 (7)	0.05588 (17)	
Cl1	0.79125 (8)	0.53542 (7)	0.59310 (8)	0.06053 (18)	
N2	0.3752 (3)	0.5613 (2)	0.1386 (4)	0.0803 (9)	
N1	0.5438 (4)	0.4614 (4)	0.2679 (4)	0.0884 (10)	
H1A	0.6204	0.4413	0.3351	0.106*	
C2	0.4639 (4)	0.3926 (3)	0.1674 (5)	0.0778 (10)	
H2	0.4800	0.3156	0.1572	0.093*	
C3	0.3583 (4)	0.4548 (3)	0.0861 (4)	0.0718 (9)	
H3	0.2849	0.4301	0.0065	0.086*	
C1	0.4905 (5)	0.5607 (4)	0.2501 (5)	0.0895 (13)	
H1	0.5276	0.6231	0.3073	0.107*	
C6	0.0239 (5)	0.6629 (5)	−0.0277 (6)	0.1064 (17)	
H6A	0.0288	0.6570	−0.1207	0.128*	0.512 (9)
H6B	−0.0676	0.6696	−0.0097	0.128*	0.512 (9)
C5A	0.1409 (7)	0.6617 (7)	0.0733 (9)	0.0645 (19)	0.488 (9)
H5A	0.1396	0.6674	0.1674	0.077*	0.488 (9)
C4A	0.2828 (8)	0.6505 (7)	0.0334 (13)	0.085 (3)	0.512 (9)
H4A1	0.3346	0.7226	0.0435	0.102*	0.512 (9)
H4A2	0.2635	0.6247	−0.0639	0.102*	0.512 (9)
C4B	0.2765 (9)	0.6630 (6)	0.1398 (10)	0.073 (2)	0.488 (9)
H4B1	0.2290	0.6558	0.2150	0.087*	0.488 (9)
H4B2	0.3340	0.7326	0.1543	0.087*	0.488 (9)
C5B	0.1662 (11)	0.6638 (8)	0.0018 (11)	0.086 (3)	0.512 (9)
H5B	0.2039	0.6651	−0.0761	0.103*	0.512 (9)

### Atomic displacement parameters (Å^2^)

**Table:**

	*U*^11^	*U*^22^	*U*^33^	*U*^12^	*U*^13^	*U*^23^
Sn1	0.04007 (11)	0.03416 (10)	0.03755 (11)	0.00060 (9)	0.00730 (7)	0.00264 (8)
Cl2	0.0611 (4)	0.0399 (3)	0.0626 (4)	−0.0058 (3)	0.0033 (3)	−0.0064 (3)
Cl3	0.0597 (4)	0.0527 (3)	0.0467 (3)	0.0001 (3)	0.0015 (3)	0.0136 (3)
Cl1	0.0553 (4)	0.0704 (4)	0.0611 (4)	0.0057 (3)	0.0250 (3)	−0.0003 (3)
N2	0.0484 (14)	0.0470 (14)	0.140 (3)	−0.0018 (11)	0.0178 (16)	0.0098 (16)
N1	0.0568 (17)	0.107 (3)	0.090 (2)	−0.0018 (19)	0.0023 (15)	0.016 (2)
C2	0.071 (2)	0.0554 (19)	0.111 (3)	0.0096 (17)	0.033 (2)	0.005 (2)
C3	0.069 (2)	0.067 (2)	0.071 (2)	−0.0158 (17)	0.0064 (16)	−0.0003 (17)
C1	0.066 (2)	0.086 (3)	0.117 (3)	−0.024 (2)	0.027 (2)	−0.038 (3)
C6	0.078 (3)	0.124 (4)	0.107 (3)	−0.015 (3)	0.009 (2)	0.037 (3)
C5A	0.064 (4)	0.076 (4)	0.059 (4)	0.012 (3)	0.028 (3)	0.014 (3)
C4A	0.068 (4)	0.068 (4)	0.127 (9)	0.020 (3)	0.042 (5)	0.045 (5)
C4B	0.075 (5)	0.052 (4)	0.081 (6)	0.000 (3)	0.005 (4)	0.002 (3)
C5B	0.099 (7)	0.078 (5)	0.072 (5)	0.010 (5)	0.010 (5)	0.023 (4)

### Geometric parameters (Å, º)

**Table:**

Sn1—Cl3	2.4131 (6)	C3—H3	0.9300
Sn1—Cl3^i^	2.4131 (6)	C1—H1	0.9300
Sn1—Cl1^i^	2.4247 (7)	C6—C5A	1.253 (8)
Sn1—Cl1	2.4247 (7)	C6—C5B	1.285 (10)
Sn1—Cl2	2.4363 (6)	C6—H6A	0.9300
Sn1—Cl2^i^	2.4363 (6)	C6—H6B	0.9300
N2—C1	1.303 (5)	C5A—C4A	1.499 (9)
N2—C3	1.349 (5)	C5A—H5A	0.9300
N2—C4B	1.517 (8)	C4A—H4A1	0.9700
N2—C4A	1.554 (8)	C4A—H4A2	0.9700
N1—C1	1.265 (6)	C4B—C5B	1.453 (9)
N1—C2	1.331 (5)	C4B—H4B1	0.9700
N1—H1A	0.8600	C4B—H4B2	0.9700
C2—C3	1.307 (5)	C5B—H5B	0.9300
C2—H2	0.9300		
			
Cl3—Sn1—Cl3^i^	180.0	C2—C3—H3	126.2
Cl3—Sn1—Cl1^i^	89.04 (3)	N2—C3—H3	126.2
Cl3^i^—Sn1—Cl1^i^	90.96 (3)	N1—C1—N2	108.8 (4)
Cl3—Sn1—Cl1	90.96 (3)	N1—C1—H1	125.6
Cl3^i^—Sn1—Cl1	89.04 (3)	N2—C1—H1	125.6
Cl1^i^—Sn1—Cl1	180.0	C5A—C6—H6A	120.0
Cl3—Sn1—Cl2	89.85 (2)	C5A—C6—H6B	120.0
Cl3^i^—Sn1—Cl2	90.15 (2)	H6A—C6—H6B	120.0
Cl1^i^—Sn1—Cl2	91.56 (3)	C6—C5A—C4A	116.0 (7)
Cl1—Sn1—Cl2	88.44 (3)	C6—C5A—H5A	122.0
Cl3—Sn1—Cl2^i^	90.15 (2)	C4A—C5A—H5A	122.0
Cl3^i^—Sn1—Cl2^i^	89.85 (2)	C5A—C4A—N2	104.8 (6)
Cl1^i^—Sn1—Cl2^i^	88.44 (3)	C5A—C4A—H4A1	110.8
Cl1—Sn1—Cl2^i^	91.56 (3)	N2—C4A—H4A1	110.8
Cl2—Sn1—Cl2^i^	180.0	C5A—C4A—H4A2	110.8
C1—N2—C3	107.2 (3)	N2—C4A—H4A2	110.8
C1—N2—C4B	111.2 (4)	H4A1—C4A—H4A2	108.9
C3—N2—C4B	137.0 (4)	C5B—C4B—N2	105.8 (7)
C1—N2—C4A	137.4 (5)	C5B—C4B—H4B1	110.6
C3—N2—C4A	113.1 (6)	N2—C4B—H4B1	110.6
C1—N1—C2	110.1 (3)	C5B—C4B—H4B2	110.6
C1—N1—H1A	124.9	N2—C4B—H4B2	110.6
C2—N1—H1A	124.9	H4B1—C4B—H4B2	108.7
C3—C2—N1	106.3 (3)	C6—C5B—C4B	129.0 (10)
C3—C2—H2	126.8	C6—C5B—H5B	115.5
N1—C2—H2	126.8	C4B—C5B—H5B	115.5
C2—C3—N2	107.6 (3)		
			
C1—N1—C2—C3	0.2 (5)	C4A—N2—C1—N1	−160.5 (6)
N1—C2—C3—N2	−0.1 (5)	C6—C5A—C4A—N2	−135.5 (8)
C1—N2—C3—C2	0.0 (5)	C1—N2—C4A—C5A	−106.0 (7)
C4B—N2—C3—C2	−152.5 (7)	C3—N2—C4A—C5A	94.2 (8)
C4A—N2—C3—C2	165.9 (4)	C1—N2—C4B—C5B	171.4 (6)
C2—N1—C1—N2	−0.1 (5)	C3—N2—C4B—C5B	−36.7 (11)
C3—N2—C1—N1	0.1 (5)	N2—C4B—C5B—C6	124.1 (9)
C4B—N2—C1—N1	160.4 (5)		

Symmetry code: (i) −*x*+2, −*y*+1, −*z*+1.

### Hydrogen-bond geometry (Å, º)

**Table:**

*D*—H···*A*	*D*—H	H···*A*	*D*···*A*	*D*—H···*A*
N1—H1*A*···Cl2	0.86	2.67	3.399 (4)	144
N1—H1*A*···Cl1	0.86	2.81	3.485 (4)	136
C2—H2···Cl3^ii^	0.93	2.79	3.672 (4)	158
C3—H3···Cl3^iii^	0.93	2.90	3.790 (4)	160
C4*A*—H4*A*1···Cl2^iv^	0.97	2.89	3.716 (9)	144
C4*A*—H4*A*1···Cl1^v^	0.97	2.94	3.743 (10)	141
C4*B*—H4*B*1···Cl1^vi^	0.97	2.98	3.702 (10)	133
C4*B*—H4*B*2···Cl1^v^	0.97	2.80	3.590 (7)	139
C5*B*—H5*B*···Cl2^vii^	0.93	2.97	3.766 (11)	145

Symmetry codes: (ii) *x*−1/2, −*y*+1/2, *z*−1/2; (iii) *x*−1, *y*, *z*−1; (iv) −*x*+3/2, *y*+1/2, −*z*+1/2; (v) *x*−1/2, −*y*+3/2, *z*−1/2; (vi) −*x*+1, −*y*+1, −*z*+1; (vii) −*x*+1, −*y*+1, −*z*.
